# Irrigation, Phosphorus Application, and Inoculation Improve the Yield and Nutritional Quality of Alfalfa by Regulating Its Photosynthetic Performance

**DOI:** 10.3390/biology15201903

**Published:** 2026-10-21

**Authors:** Huijuan Huo, Yanliang Sun, Kongqin Wei, Dongjie Xia, Qianbing Zhang

**Affiliations:** 1 College of Animal Science and Technology, Shihezi University, Shihezi 832003, China; huohuijuan2023@163.com (H.H.);; 2 Bingtuan Key Laboratory for Efficient Utilization of Non-Grain Feed Resources, College of Animal Science and Technology, Shihezi University, Shihezi 832003, China

**Keywords:** *Medicago sativa* L., drought stress, phosphorus fertilizer, phosphate-solubilizing bacteria, production performance, photosynthetic performance

## Abstract

Water and phosphorus (P) scarcity severely limit the high-quality production of alfalfa (*Medicago sativa* L.) in arid regions. To clarify the synergistic regulatory effects of irrigation, P application, and phosphate-solubilizing bacteria (PSB) inoculation, this study used a three-factor completely randomized design. Three irrigation levels (W1: 40–45%, W2: 55–60%, W3: 70–75% of field capacity), two P application levels (P1: 50 mg kg^−1^, P2: 100 mg kg^−1^), and two inoculation treatments (J0: no inoculation, J1: inoculation with *Bacillus mucilaginosus*) were set to explore their interactive effects on photosynthesis, growth, and nutritional quality of alfalfa. The results showed that under mild drought, high-P combined with PSB inoculation significantly increased plant height (PH), chlorophyll content (Chl), and leaf instantaneous water-use efficiency (WUEi) while maintaining stable dry matter yield (DM). This treatment also increased crude protein (CP) content and relative feed value (RFV), and decreased neutral and acid detergent fiber contents, thus improving forage quality. In conclusion, under mild drought (55–60% of field capacity), P fertilization at 100 mg·kg^−1^ combined with PSB inoculation enhances photosynthetic performance, water- and phosphorus-use efficiencies, and improves yield and quality. It provides a theoretical reference for water-saving and high-quality alfalfa production in arid regions.

## Simple Summary

In recent years, although studies on the high-yield and high-quality cultivation techniques of alfalfa under drought stress have been increasing, there is still a research gap in the interaction mechanism among irrigation, phosphorus fertilizer, and phosphate-solubilizing bacteria (PSB), as well as their synergistic effects in alleviating drought, improving production performance, and nutritional quality. This study clarified the synergistic effect of water–phosphorus–bacteria. The results showed that applying high phosphorus combined with PSB under mild drought conditions improved photosynthetic performance, water use efficiency, and nutritional quality. The mechanism is that PSB activates insoluble phosphorus in the soil and synergistically regulates photosynthetic performance with water and phosphorus, which not only alleviates the inhibition of drought stress but also optimizes the distribution of photosynthetic products.

## 1. Introduction

Alfalfa (*Medicago sativa* L.), a high-quality perennial leguminous forage grass, is known as the “King of Forages” due to its high yield potential, excellent nutritional value, superior palatability, and remarkable ecological effects, and plays an irreplaceable role in global agricultural production [1]. Continued global warming exacerbates drought stress in arid and semi-arid regions. As a high water-demanding crop, alfalfa relies on irrigation in many cultivation areas to ensure its normal growth [2]. The main production areas of alfalfa in China are located in the northern arid and semi-arid regions, where a severe shortage of water resources leads to its growth being highly susceptible to drought stress [3]. The impact of drought stress on alfalfa’s yield and quality has become a major limiting factor in its production and a core challenge to the sustainable development of the alfalfa industry in this region [4]. Therefore, it is crucial to explore effective strategies to alleviate the constraints of drought stress on the efficient production of alfalfa. As a key nutrient element regulating plant growth and development, phosphorus (P) not only participates in many physiological processes such as photosynthesis but also plays an important role in yield formation [5]. However, the dominant soil type in the Shihezi area of Xinjiang is grey desert soil, which is characterized by low available phosphorus (AP) content. Studies have shown that P in soil has poor mobility and is easily fixed by metal ions to form insoluble compounds, resulting in reduced bioavailability of soil P and thereby limiting crop absorption of P and their normal growth and development [6]. Drought stress reduces the diffusion rate of nutrients in the rhizosphere and intensifies P fixation, thereby further limiting rhizosphere P supply and the uptake and utilization of P by plant roots [7]. Soil P deficiency can significantly restrict plant growth and productivity [8]; conversely, the over-application of P fertilizer not only results in resource waste but also may deteriorate soil physical and chemical properties, thereby impeding the normal growth and development of plants [9].

Phosphate-solubilizing bacteria (PSB) can modify the pH of the rhizosphere microenvironment by secreting organic acids, protons, and other substances, thereby dissolving fixed, insoluble phosphates in the soil and increasing the level of AP in the rhizosphere. This process can alleviate P supply limitations to some extent [9]. Studies have shown that the drought tolerance of mycorrhizal plants is closely related to the levels of malondialdehyde (MDA) and soluble sugar (SS) in their bodies. PSB inoculation can effectively enhance plant drought resistance by significantly reducing MDA concentration and increasing SS content, thereby regulating these two key physiological indicators [10]. Photosynthesis is a core physiological process in plants and plays a crucial role in crop yield formation [11]. As key pigments for photosynthesis, chlorophylls govern the absorption, transmission, and transformation of light energy in plant leaves, and their content significantly regulates plant growth, development, and yield [12]. Studies have shown that plant photosynthesis is highly responsive to changes in environmental factors. Drought stress can inhibit photosynthetic carbon assimilation and biomass accumulation by inducing stomatal closure, limiting CO_2_ diffusion, and disrupting photosynthetic metabolic processes [13]. Under mild and moderate drought stress, the decrease in photosynthesis is mainly dominated by stomatal limitation; whereas under severe drought stress, with damage to chloroplast ultrastructure, the reduction in photosynthesis is mainly attributed to the non-stomatal limitation of chloroplast CO_2_ fixation capacity [14]. P is a key element involved in chloroplast structural components, the photosynthetic electron transport chain, and ATP synthesis. Therefore, soil P availability directly determines the operational efficiency of photosynthetic energy metabolism and carbon assimilation processes [15]. Additional studies have indicated that water stress reduces biomass accumulation in alfalfa by inhibiting photosynthesis, while P application can effectively alleviate this negative effect. By promoting alfalfa growth under water stress, P fertilization ultimately enhances dry matter yield (DM) [16]. Another study showed that plant growth-promoting rhizobacteria (PGPR) effectively inhibit chlorophyll degradation under drought stress by improving plant water status and increasing relative leaf water content, thereby maintaining the photosynthetic capacity of alfalfa seedlings, enhancing their drought resistance, and ensuring normal growth and development [17]. Studies have demonstrated that under drought-stress conditions, appropriate phosphorus fertilization can offset alfalfa yield losses. Compared with irrigation measures, phosphorus fertilization produces more pronounced improvements in alfalfa drought tolerance by enhancing yield, water-use efficiency, and crude-protein content [18]. Co-inoculation with arbuscular mycorrhizal fungi and PSB, combined with the application of 100 mg kg^−1^ P fertilizer, can effectively promote alfalfa growth and improve its nutritional quality [19].

Extensive research has been conducted on water-saving irrigation and nutrient regulation in alfalfa, and previous studies have shown that appropriate P application or inoculation with PSB can enhance drought tolerance and productivity in alfalfa. However, most existing studies have focused on single-factor effects or two-factor interactions, such as water–P and bacteria–P interactions [20,21], whereas the integrated regulatory effects of the three-way interaction among irrigation, P fertilizer, and PSB remain insufficiently understood. Therefore, taking alfalfa as the research material, this study established different irrigation, phosphorus-fertilizer, and PSB-inoculation treatments under pot-culture conditions. From the perspectives of plant growth, photosynthetic physiology, yield components, and forage quality, we comprehensively analyzed the physiological basis underlying the synergistic regulation of alfalfa productive performance by irrigation, phosphorus fertilizer, and PSB inoculation. We further clarified the synergistic regulatory mechanisms of phosphorus fertilizer and PSB under drought stress, and screened suitable combinations of irrigation, phosphorus-fertilizer application, and PSB inoculation. This work aims to improve the theoretical basis for the coordinated regulation of water–phosphorus–bacteria interactions and quality improvement of alfalfa under drought conditions, and provide theoretical support for high-quality alfalfa production and subsequent field-validation trials. Based on this, the following hypotheses were proposed: (1) Drought stress inhibits the normal growth and development of alfalfa, whereas the combined application of appropriate P fertilizer and PSB can improve P availability and plant physiological status, thereby alleviating the adverse effects of drought stress on plant growth. (2) The three-way interaction among irrigation, P fertilizer, and PSB can maintain high photosynthetic capacity in alfalfa by regulating stomatal conductance, photosynthetic pigment accumulation, and gas exchange processes. (3) Appropriate combinations of irrigation, P fertilizer, and PSB inoculation can enhance the physiological adaptability of alfalfa to drought stress, thereby mitigating the adverse effects of drought stress on plant growth, DM, and nutritional quality.

## 2. Materials and Methods

### 2.1. Test Materials

The tested alfalfa cultivar was “Xinmu No. 4”. The PSB strain used in the experiment was *Bacillus mucilaginosus*, provided by the Agricultural Culture Collection of China (ACCC) with the strain number 10011. The strain was cultured on potassium bacterial agar medium, with the following formulation: sucrose 10 g, K_2_HPO_4_ 0.5 g, yeast extract 0.4 g, MgSO_4_·7H_2_O 0.2 g, MgCl_2_ 0.2 g, agar 15 g, distilled water 1 L, and pH 7.0–7.2. The phosphorus-free Hoagland nutrient solution was a modified nutrient solution without phosphate-containing components or calcium nitrate. Its composition was as follows: potassium sulfate 607 mg L^−1^, magnesium sulfate 493 mg L^−1^, ammonium sulfate 66.02 mg L^−1^, EDTA-Fe sodium salt 20 mg L^−1^, ferrous sulfate 15 mg L^−1^, boric acid 2.86 mg L^−1^, borax 4.5 mg L^−1^, manganese sulfate 2.13 mg L^−1^, copper sulfate 0.05 mg L^−1^, and zinc sulfate 0.22 mg L^−1^. The tested soil was collected from No.2 Experimental Station of Shihezi University (44°33′ N, 86°00′ E). After being sieved through a 0.5 cm mesh sieve, it was autoclaved at 121 °C for 30 min for subsequent use. The basic physical and chemical properties of the soil are detailed in Table 1. The details of each experimental treatment are presented in Table 2.

### 2.2. Experimental Design

The experiment adopted a three-factor completely randomized design, with three factors set: irrigation, P application, and inoculation. Three levels were set for irrigation treatments: moderate drought (W1, 40–45% field capacity, FC), mild drought (W2, 55–60% FC), and normal irrigation (W3, 70–75% FC). The P application treatments consisted of two levels: 50 mg kg^−1^ P_2_O_5_ (P1) and 100 mg kg^−1^ P_2_O_5_ (P2). For inoculation treatments, two levels were designed: no inoculation (J0) and inoculation (J1). The experiment consisted of 12 treatments, with each individual pot serving as an independent experimental unit. Each treatment included 12 pots, corresponding to 12 biological replicates. *Bacillus mucilaginosus* was inoculated on potassium bacterial agar medium and incubated at a constant temperature of 37 °C for 5 days. The concentration of the bacterial suspension was adjusted to 1 × 10^8^ cfu mL^−1^ using the dilution plate method, which served as the inoculum. At the trifoliate stage of alfalfa, 10 mL of the bacterial suspension was applied to each pot in the inoculation treatment group (J1), yielding a final viable-cell inoculation dose of 1 × 10^9^ cfu pot^−1^. For the non-inoculation treatment group (J0), an equal volume of autoclaved bacterial suspension was applied to the soil along with irrigation water. The experiment was conducted at the Experimental Station of the College of Animal Science and Technology, Shihezi University, as an outdoor rain-sheltered pot experiment. Plants were grown under natural light throughout the experiment, and white plastic tarpaulins were used only during rainfall events to eliminate the interference of natural precipitation with the irrigation treatments. Environmental conditions during the 2024 and 2025 experimental periods were obtained from contemporaneous meteorological records at the experimental station. The annual mean air temperatures in the experimental area were 9 °C and 10 °C, respectively; the mean relative humidity values were 36% and 37.5%, respectively; the natural photoperiods were approximately 9–12 h d^−1^ and 9–11 h d^−1^, respectively; and the variation ranges of photosynthetic photon flux density (PPFD) were 1100–1300 and 1000–1200 μmol m^−2^ s^−1^, respectively.

The pots used in the experiment had a top diameter of 23 cm, a bottom diameter of 15 cm, and a height of 17 cm. Prior to use, the pots were cleaned and disinfected with 75% ethanol. The experimental substrate was composed of sterilized soil and perlite (to prevent soil compaction) mixed at a volume ratio of 3:1, and each pot was filled with 3 kg of the sterilized mixed substrate. Field capacity in this experiment was determined based on the mixed substrate. Before the experiment commenced, the core-ring soaking method was used to measure the field capacity of the tested mixed substrate. These values were used as the basis to set the normal irrigation, mild drought, and moderate drought treatments, and to calculate the target pot weight corresponding to each irrigation treatment for water control throughout the experimental period. On 19 May 2024, high-quality alfalfa seeds with good plumpness and uniform size were selected for sowing. Ten seeds were sown per pot and covered with soil to a depth of 1–2 cm. The pot experiment was conducted at the Experimental Station of the College of Animal Science and Technology, Shihezi University. When the seedlings grew to the three-leaf stage, thinning was performed, and 5 robust seedlings with uniform growth vigor were retained per pot. Starting from the three-leaf stage, each pot was quantitatively irrigated with 200 mL of phosphorus-free Hoagland’s nutrient solution every 14 days, which ceased after the application of P fertilizer. P fertilizer was applied at the branching stage, with triple superphosphate (P_2_O_5_ ≥ 45%) of good water solubility selected as the phosphorus source. The application rate was calculated based on its available P_2_O_5_ content and applied as a single dose. The specific fertilization dates were 21 July 2024 and 3 June 2025, respectively. Before the branching stage of alfalfa, soil moisture in all treatments was maintained at field water-holding capacity to ensure normal seedling growth. Water regulation was initiated from the branching stage onward. Each day between 19:00 and 20:00, water was replenished using the weighing method to maintain soil moisture in each treatment within 40–75% of field water-holding capacity. During water replenishment, water losses caused by soil evaporation and plant transpiration were uniformly corrected based on the weighing results. Alfalfa was harvested at the initial flowering stage, with the specific harvest dates being 17 August 2024 and 18 June 2025, respectively.

### 2.3. Determination Indicators and Methods

Each treatment had a total of 12 pots serving as biological replicates. Plant height and stem diameter are non-destructive measurement indices. To ensure experimental uniformity, 10 pots with healthy and uniform growth were selected for these measurements. Destructive cutting-harvest sampling was required for hay yield determination. Three pots with homogeneous growth were randomly selected from the 12 pots to measure hay yield, and these three pots were excluded from subsequent leaf sampling. Leaf-sampling-based assays for photosynthetic pigments and photosynthetic parameters were performed using the remaining nine pots. Accordingly, indices including hay yield, nutritional quality, photosynthetic pigments, and photosynthetic parameters were not derived from the same set of potted plants.

#### 2.3.1. Growth Performance

Plant height (PH): At the initial flowering stage, 10 pots were randomly selected from each treatment as biological replicates. The absolute height of alfalfa was measured using a measuring tape, and the mean value was calculated. Simultaneously, stem diameter (SD) was measured at 5 cm above the soil surface using a digital vernier caliper (Ningbo Deli Tools Co., Ltd., Ningbo, China)with an accuracy of 0.01 cm, and the average value was recorded. DM: The determination of hay yield involved destructive above-ground harvesting. On the same day as the plant height measurements, three pots of alfalfa with similar growth were selected from each treatment as biological replicates. The plants were cut at a standard stubble height of 5 cm, and the fresh weight was measured. Subsequently, the samples were oven-dried at 105 °C for 30 min to deactivate enzymes and then dried at 65 °C to a constant weight. The dry matter weight was recorded, and the average value was calculated. Stem-to-leaf ratio (S/L): The oven-dried alfalfa samples were manually separated into stems and leaves. The mass of stems and leaves from each sample was weighed separately to calculate the stem-to-leaf ratio, and the average value was determined.

#### 2.3.2. Photosynthetic Pigment Content and Soluble Sugar Content

At the time of yield determination, three pots were randomly selected from each treatment as biological replicates, and fresh, intact middle leaflets from the upper one-third of the alfalfa plants were collected. The chlorophyll (Chl) and carotenoid (Car) contents in the fresh alfalfa leaves were determined using a spectrophotometric method [22]. The SS content was measured via the anthrone colorimetric method.

#### 2.3.3. Photosynthetic Characteristics

Prior to the alfalfa harvest, photosynthetic parameters were measured on clear, cloudless days between 11:00 and 13:00 h to minimize interference caused by diurnal variations in environmental factors. Measurements were performed under outdoor natural conditions; the reference CO_2_ concentration, leaf-chamber temperature, and relative humidity were not artificially controlled and fluctuated dynamically with the ambient atmospheric environment. From each treatment, three pots were selected as biological replicates, and intact leaves at the middle leaf position from the upper one-third of the alfalfa plants were collected. When the light intensity reached 1500 μmol m^−2^ s^−1^, a portable photosynthesis system (LI-6400XT, LI-COR Biosciences, Lincoln, NE, USA) was employed to determine key photosynthetic parameters, including the net photosynthetic rate (Pn), transpiration rate (Tr), stomatal conductance (Gs), and intercellular CO_2_ concentration (Ci). The average value obtained from three leaves sampled within the same pot was taken as the photosynthetic-parameter value for that pot. The instantaneous water use efficiency (WUEi) and stomatal limitation value (Ls) were subsequently calculated using the following formulas (in Equation (2), Ca represents the atmospheric CO_2_ concentration):

WUEi = Pn/Tr (1)

Ls = 1 − Ci/Ca (2)

#### 2.3.4. Nutritional Quality

The dried forage samples were ground and passed through a 0.5 mm sieve, then stored in labeled zip-lock bags for subsequent analysis of their nutritional composition. The measured value of each individual pot was treated as an independent experimental unit, and data from the three pots were used as biological replicates for statistical analysis. Crude protein (CP) content was determined using the Kjeldahl method. The contents of neutral detergent fiber (NDF) and acid detergent fiber (ADF) were analyzed according to the Van Soest method. Relative feed value (RFV) was calculated based on NDF and ADF contents using the following formula:

RFV = (88.9 − 0.779 × ADF) × (120/NDF)/1.29 (3)

### 2.4. Data Statistical Analysis

The data were organized using Microsoft Excel 2021 and statistically analyzed using IBM SPSS Statistics 26.0. The alfalfa pot-cultured plants in this experiment were established in 2024, and measurements were continuously conducted on the same potted plants in 2025. The data from the two years did not originate from independent experimental replicates. Therefore, year was not treated as an experimental factor in this study, and analysis of variance (ANOVA) was performed separately for the datasets of 2024 and 2025. Meanwhile, the overall variation trends of each measured indicator were largely consistent between the two years, with no obvious year-specific differences. Accordingly, all sample data from both years were integrated for Pearson correlation analysis and the Mantel test, aiming to more comprehensively reveal the intrinsic relationships among the measured indicators. A three-way ANOVA was used to test the main effects of irrigation level (W), phosphorus application level (P), and microbial inoculation treatment (J), as well as their interaction effects, including W × P, W × J, P × J, and W × P × J. All statistical analyses were performed by ANOVA, using measurements from individual pots as independent biological replicates. Before ANOVA, normality and homogeneity of variance were tested separately for each year. Tukey’s HSD multiple comparison tests were performed within the same year and for each individual indicator, using the 12 W × P × J treatment combinations as the comparison groups to evaluate differences among treatment combinations. The significance level was set at *p* < 0.05. The figures and tables were generated using Origin 2021 and the ChiPlot online platform (https://www.chiplot.online/, accessed on 8 January 2025).

## 3. Results

### 3.1. Growth Traits of Alfalfa Under Water–Phosphorus–Bacteria Interaction

**Figure 1:**
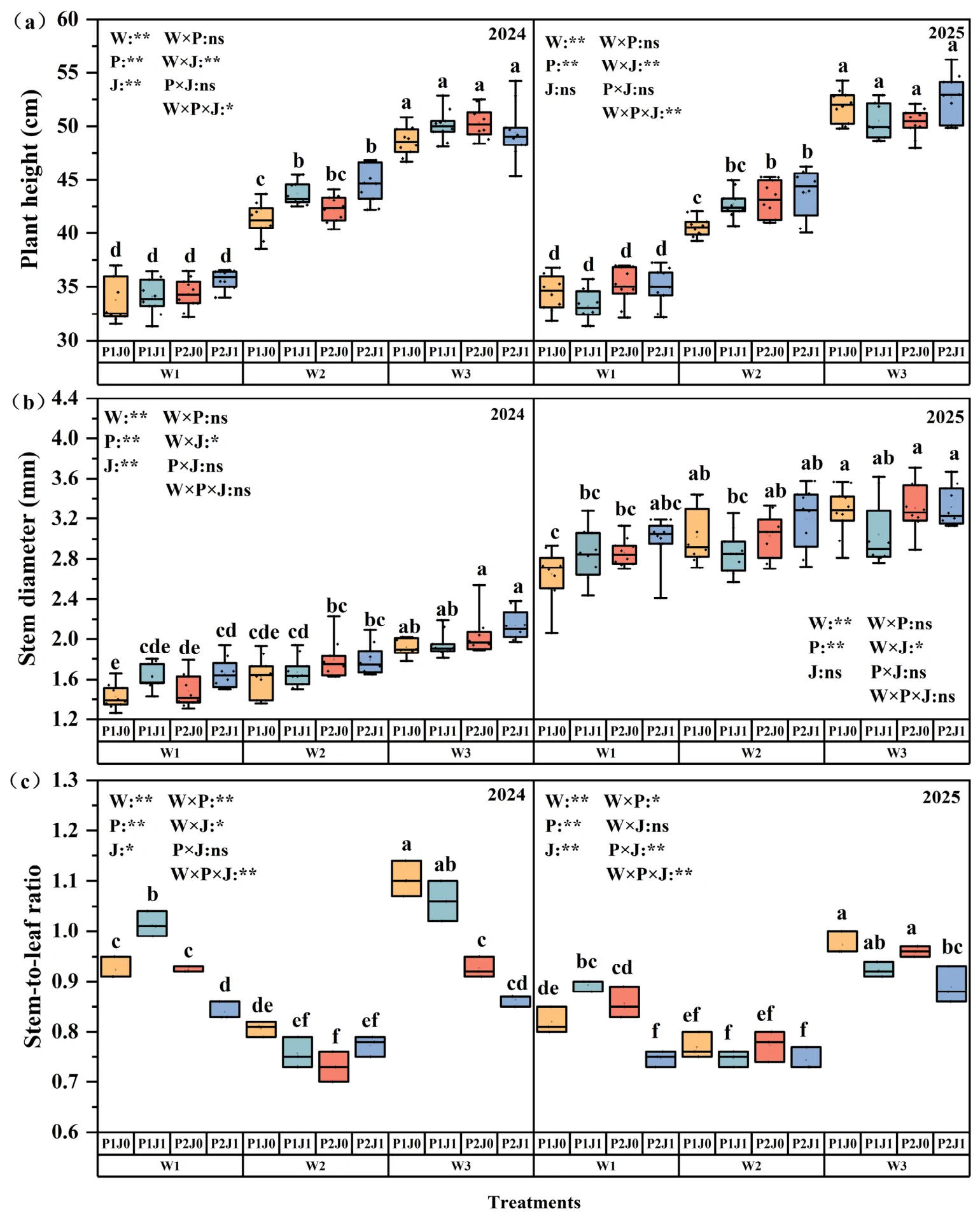
Growth traits of alfalfa under different treatments (2024–2025). (**a**–**c**) PH, SD, and S/L of alfalfa under different treatments, respectively. Data are presented as mean ± standard error, with *n* = 10 for PH and SD, and *n* = 3 for S/L. The four different colored bars within each water treatment represent P1J0, P1J1, P2J0 and P2J1, respectively. The small dots inside the colored blocks represent individual replicate observations. Different lowercase letters indicate significant differences among treatments (*p* < 0.05), while ns denotes no significant difference (*p* > 0.05). * and ** denote significant differences at *p* < 0.05 and *p* < 0.01 levels, respectively. W1, W2, and W3 represent moderate drought (40–45% field capacity), mild drought (55–60% field capacity), and normal irrigation (70–75% field capacity), respectively. P1 and P2 represent low P (50 mg kg^−1^ P_2_O_5_) and high P (100 mg kg^−1^ P_2_O_5_), respectively, while J0 and J1 represent non-inoculation (0 mL pot^−1^) and inoculation with PSB (10 mL pot^−1^), respectively.

Irrigation, P fertilizer, and PSB had highly significant effects on PH, SD, and S/L of alfalfa (*p* < 0.01). The interaction between irrigation and PSB significantly influenced PH and SD (*p* < 0.05), while the three-way interaction among irrigation, P fertilizer, and PSB had a highly significant effect on PH and S/L (*p* < 0.01) (Figure 1, Table S1). Results from both years showed that under the same interaction conditions of P fertilizer and PSB, with the intensification of drought stress, the PH and SD of alfalfa showed a gradual decreasing trend, while the S/L exhibited a trend of first decreasing and then increasing. Under mild drought stress, alfalfa PH in the high-P combined with PSB inoculation treatment was significantly higher than that of other treatments (Figure 1a). Compared with the low-P treatment without PSB inoculation under normal irrigation, the high-P treatment with PSB inoculation under mild drought stress reduced the SD of alfalfa, yet the difference was non-significant (Figure 1b). Compared with the low-P treatment without PSB inoculation, the high-P treatment with PSB inoculation significantly decreased the S/L of alfalfa under mild and moderate drought stresses (*p* < 0.05), with a reduction range of 3.90% to 8.70%. Under normal irrigation, the low-P treatment without PSB inoculation exhibited the highest S/L (Figure 1c).

### 3.2. Photosynthetic Pigments and Soluble Sugar Content in Alfalfa Under Water–Phosphorus–Bacteria Interaction

**Figure 2:**
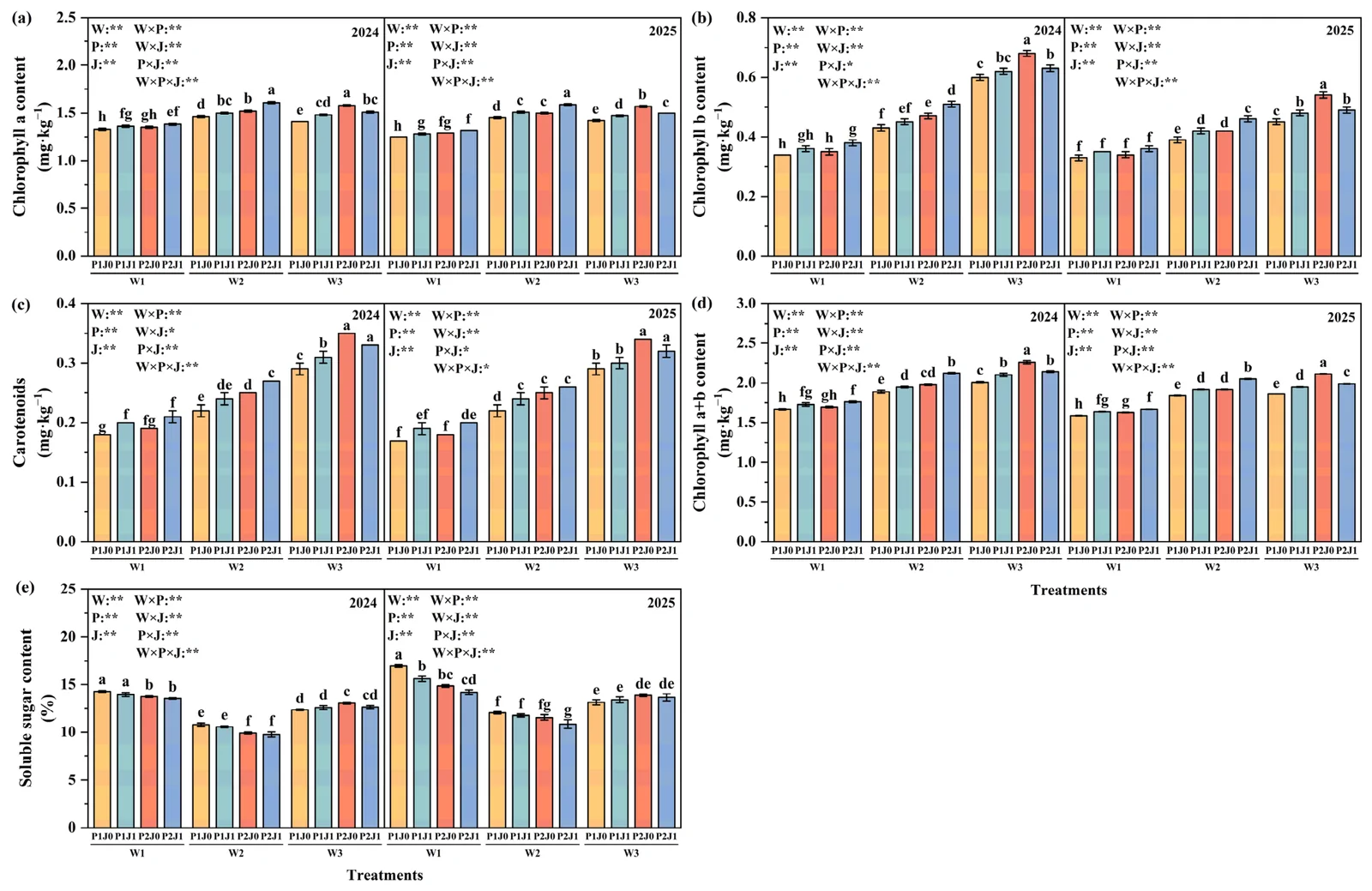
Photosynthetic pigment and soluble sugar contents of alfalfa under different treatments (2024–2025). (**a**–**e**) Chl a content, Chl b content, Car content, Chl (a + b) content, and SS content in alfalfa leaves under different treatments, respectively. Data are presented as mean ± standard error (*n* = 3). The four different colored bars within each water treatment represent P1J0, P1J1, P2J0 and P2J1, respectively. Different lowercase letters indicate significant differences among treatments (*p* < 0.05), * and ** denote significant differences at *p* < 0.05 and *p* < 0.01 levels, respectively. W1, W2, and W3 represent moderate drought (40–45% field capacity), mild drought (55–60% field capacity), and normal irrigation (70–75% field capacity), respectively. P1 and P2 represent low P (50 mg kg^−1^ P_2_O_5_) and high P (100 mg kg^−1^ P_2_O_5_), respectively, while J0 and J1 represent non-inoculation (0 mL pot^−1^) and inoculation with PSB (10 mL pot^−1^), respectively.

Irrigation, P fertilizer, PSB, the interaction between irrigation and P, the interaction between irrigation and PSB, and the three-factor interaction of irrigation, P, and PSB all had highly significant effects on the chlorophyll a (Chl a) content, chlorophyll b (Chl b) content, carotenoids (Car) content, total chlorophyll (Chl (a + b)) content, and SS content of alfalfa (*p* < 0.01) (Figure 2, Table S2). Results from both years showed that under the same interaction conditions of P fertilizer and PSB, with the intensification of drought stress, the Chl a content of alfalfa showed a trend of first increasing and then decreasing, while the SS content exhibited a trend of first decreasing and then increasing. The maximum chlorophyll a concentration across both experimental years was observed in the high-P treatment with PSB inoculation under mild drought stress. Compared with the low-P treatment without PSB inoculation under normal irrigation, the high-P treatment with PSB inoculation under mild drought stress significantly increased Chl a content and Chl (a + b) content of alfalfa in both 2024 and 2025 (*p* < 0.05), with growth increments ranging from 11.97% to 14.18% and 5.47% to 10.22%, respectively (Figure 2a,d). Under normal irrigation conditions, the highest Chl b and Car contents were recorded in the high-P treatment without PSB inoculation (Figure 2b,c). The maximum SS content across both experimental years was detected in the low-P treatment without PSB inoculation under moderate drought stress. In contrast, the high-P treatment with PSB inoculation under moderate drought stress significantly reduced SS content in alfalfa (*p* < 0.05), with a decrease ranging from 5.18% to 16.31% (Figure 2e).

### 3.3. Photosynthetic Characteristics of Alfalfa Under Water–Phosphorus–Bacteria Interaction

**Figure 3:**
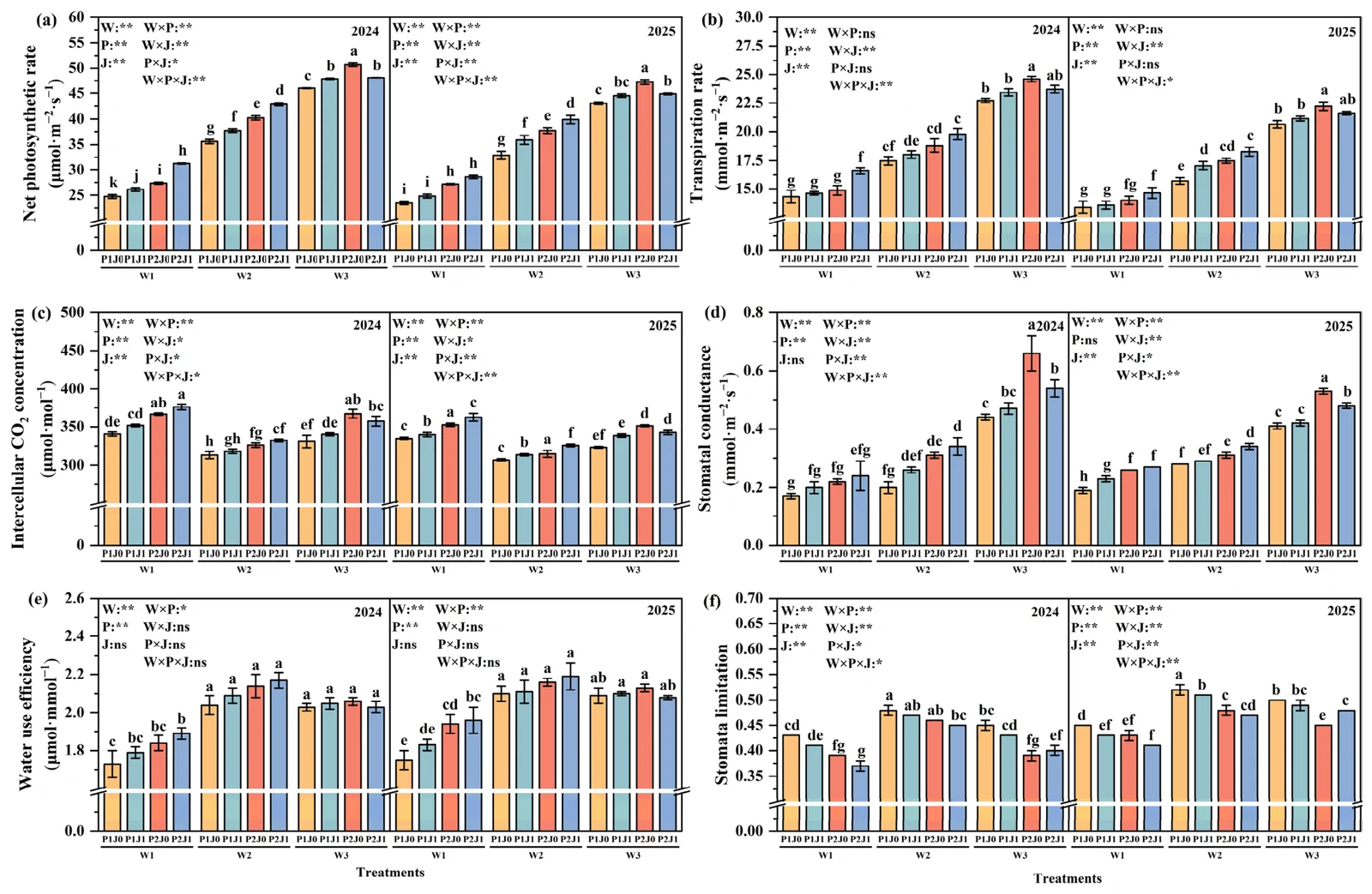
Photosynthetic characteristics of alfalfa under different treatments (2024–2025). (**a**–**f**) Pn, Tr, Ci, Gs, WUEi, and Ls in alfalfa leaves under different treatments, respectively. Data are presented as mean ± standard error (*n* = 3). The four different colored bars within each water treatment represent P1J0, P1J1, P2J0 and P2J1, respectively. Different lowercase letters indicate significant differences among treatments (*p* < 0.05), while ns denotes no significant difference (*p* > 0.05). * and ** denote significant differences at *p* < 0.05 and *p* < 0.01 levels, respectively. W1, W2, and W3 represent moderate drought (40–45% field capacity), mild drought (55–60% field capacity), and normal irrigation (70–75% field capacity), respectively. P1 and P2 represent low P (50 mg kg^−1^ P_2_O_5_) and high P (100 mg kg^−1^ P_2_O_5_), respectively, while J0 and J1 represent non-inoculation (0 mL pot^−1^) and inoculation with PSB (10 mL pot^−1^), respectively.

Irrigation, P fertilizer, PSB, the interaction between irrigation and PSB, and the three-way interaction of irrigation, P, and PSB all exerted highly significant effects on the Pn, Tr, Gs, Ci, and Ls of alfalfa (*p* < 0.01). Meanwhile, irrigation, P fertilizer, and their two-way interaction exerted highly significant (*p* < 0.01) effects on WUEi of alfalfa leaves (*p* < 0.01) (Figure 3, Table S3). Results from both years showed that under the same interaction conditions of P fertilizer and PSB, with the intensification of drought stress, the Pn, Tr, and Gs of alfalfa showed a gradually decreasing trend across both years, while the Ci concentration exhibited a trend of decreasing and then increasing, and the leaf WUEi and Ls both showed a trend of first increasing and then decreasing. Under normal irrigation conditions, all photosynthetic parameters of alfalfa remained at relatively high levels overall, among which the high-P treatment without PSB inoculation significantly elevated the Pn, Tr, and Gs of alfalfa (*p* < 0.05) (Figure 3a,b,d). Under the high-P condition with PSB inoculation, the Ci under moderate drought stress was significantly higher than that under normal irrigation (*p* < 0.05) (Figure 3c). The leaf WUEi reached its maximum value in the high-P treatment with PSB inoculation under mild drought stress; mild drought stress significantly elevated the leaf WUEi of alfalfa relative to moderate drought stress (*p* < 0.05) (Figure 3e). Under normal irrigation without PSB inoculation, the Ls in the low-P treatment was significantly higher than that in the high-P treatment (*p* < 0.05). Under low-P supply without PSB inoculation, the Ls under mild drought stress was significantly higher than that under moderate drought stress (*p* < 0.05) (Figure 3f).

### 3.4. Nutritional Quality of Alfalfa Under Water–Phosphorus–Bacteria Interaction

**Figure 4:**
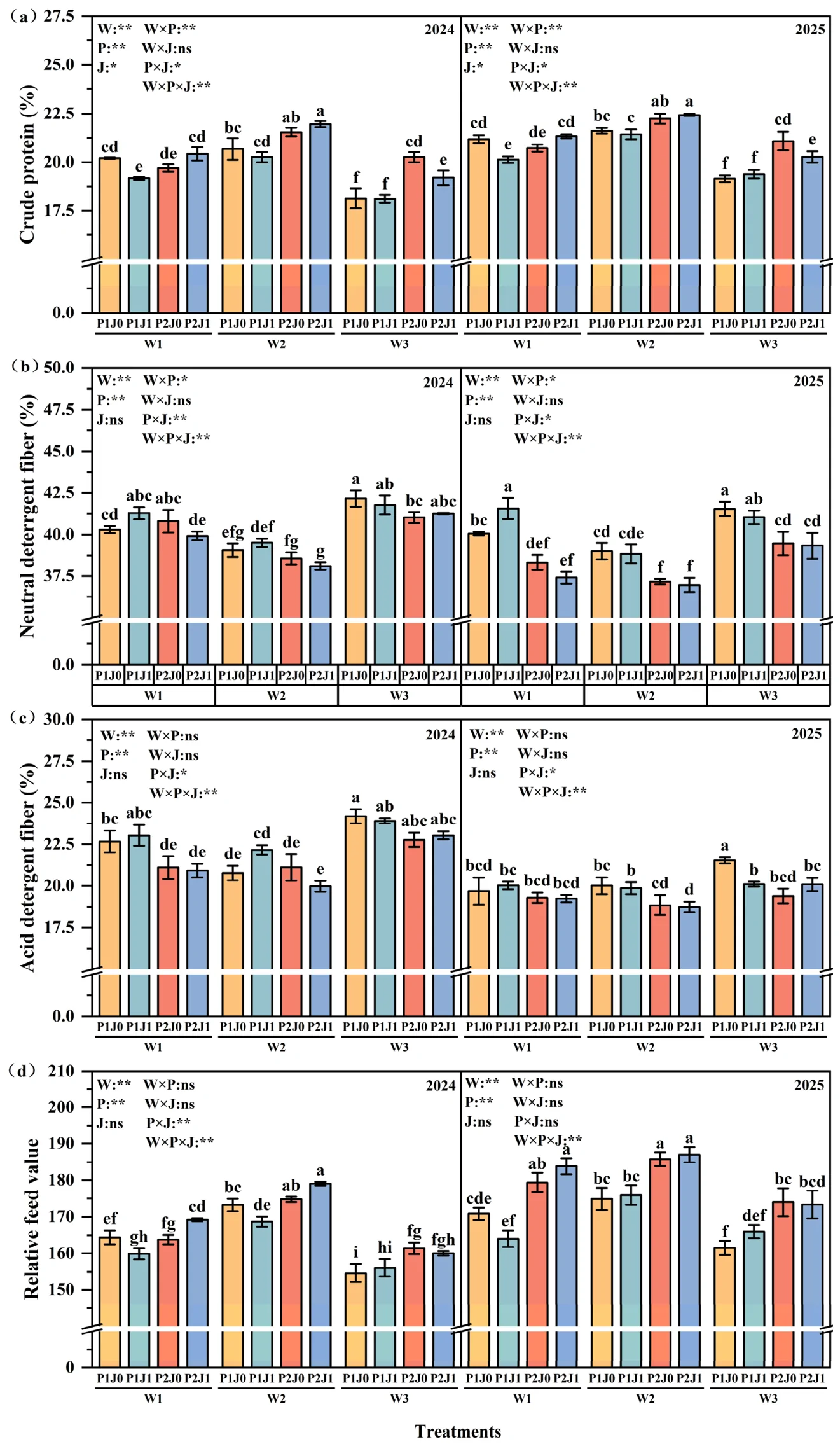
Nutritional quality of alfalfa under different treatments (2024–2025). (**a**–**d**) CP content, NDF content, ADF content, and RFV of alfalfa under different treatments, respectively. Data are presented as mean ± standard error (*n* = 3). The four different colored bars within each water treatment represent P1J0, P1J1, P2J0 and P2J1, respectively. Different lowercase letters indicate significant differences among treatments (*p* < 0.05), while ns denotes no significant difference (*p* > 0.05). * and ** denote significant differences at *p* < 0.05 and *p* < 0.01 levels, respectively. W1, W2, and W3 represent moderate drought (40–45% field capacity), mild drought (55–60% field capacity), and normal irrigation (70–75% field capacity), respectively. P1 and P2 represent low P (50 mg kg^−1^ P_2_O_5_) and high P (100 mg kg^−1^ P_2_O_5_), respectively, while J0 and J1 represent non-inoculation (0 mL pot^−1^) and inoculation with PSB (10 mL pot^−1^), respectively.

Irrigation, P fertilizer, and the three-way interaction of irrigation, P fertilizer, and PSB had highly significant effects on the CP content, NDF content, ADF content, and RFV of alfalfa (*p* < 0.01) (Figure 4, Table S4). Results from both years showed that under the same interaction conditions of P fertilizer and PSB, with the intensification of drought stress, the CP content and RFV of alfalfa showed a trend of first increasing and then decreasing, while the NDF content and ADF content exhibited a trend of first decreasing and then increasing. The maximum values of CP content and RFV, as well as the minimum values of NDF content and ADF content of alfalfa, were all observed in the high-P treatment with PSB inoculation under mild drought stress (Figure 4). Under normal irrigation and high-P conditions, the treatment without PSB inoculation significantly increased CP content compared with the treatment with PSB inoculation (*p* < 0.05) (Figure 4a). Compared with treatments under normal irrigation, the high-P treatment with PSB inoculation under mild drought stress significantly elevated CP content and RFV of alfalfa (*p* < 0.05), with growth increments ranging from 6.02% to 17.49% and 6.95% to 13.65%, respectively. Meanwhile, this treatment significantly reduced NDF content and ADF content (*p* < 0.05), with decreases ranging from 6.00% to 11.00% and 3.40% to 17.45%, respectively (Figure 4).

### 3.5. Dry Matter Yield of Alfalfa Under Water–Phosphorus–Bacteria Interaction

**Figure 5:**
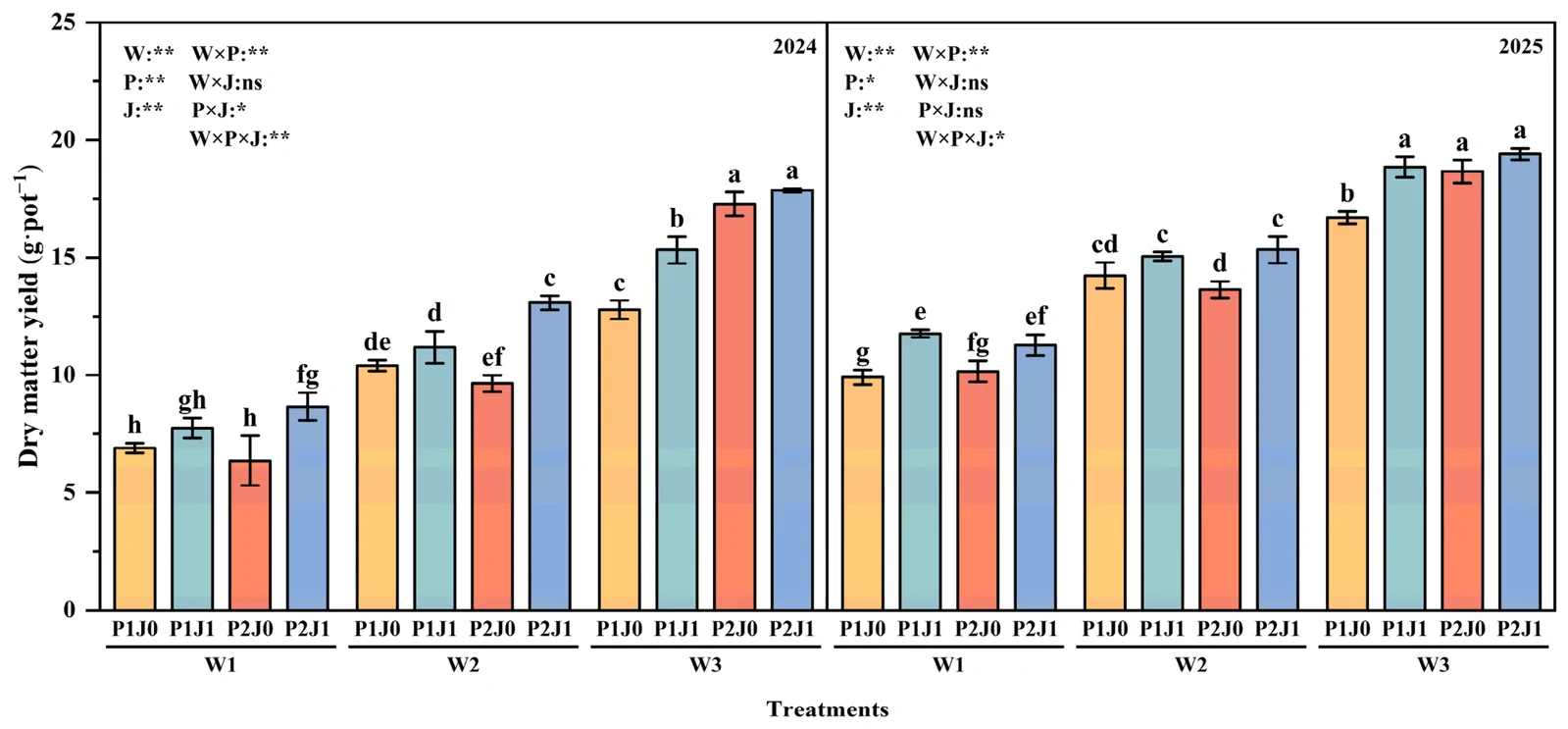
Dry matter yield of alfalfa under different treatments (2024–2025). Data are presented as mean ± standard error (*n* = 3). The four different colored bars within each water treatment represent P1J0, P1J1, P2J0 and P2J1, respectively. Different lowercase letters indicate significant differences among treatments (*p* < 0.05), while ns denotes no significant difference (*p* > 0.05). * and ** denote significant differences at *p* < 0.05 and *p* < 0.01 levels, respectively. W1, W2, and W3 represent moderate drought (40–45% field capacity), mild drought (55–60% field capacity), and normal irrigation (70–75% field capacity), respectively. P1 and P2 represent low P (50 mg kg^−1^ P_2_O_5_) and high P (100 mg kg^−1^ P_2_O_5_), respectively, while J0 and J1 represent non-inoculation (0 mL pot^−1^) and inoculation with PSB (10 mL pot^−1^), respectively.

Irrigation, P fertilizer, PSB, the two-way interaction between irrigation and P, and the three-way interaction among irrigation, P, and PSB all exerted highly significant effects on the DM of alfalfa (*p* < 0.01) (Figure 5, Table S1). Results from both years showed that under the same interaction conditions of P fertilizer and PSB, with the intensification of drought stress, the DM of alfalfa showed a gradually decreasing trend. DM under each treatment was generally maintained at a low level under moderate drought stress. In 2024, the DM of the high-P treatment with PSB inoculation under mild drought stress was higher than that of the low-P treatment without PSB inoculation under normal irrigation, whereas consistent treatment differences were not detected in 2025. Under mild drought stress with high-P supply, PSB inoculation significantly improved the DM of alfalfa (*p* < 0.05). Compared with the low-P treatment without PSB inoculation under normal irrigation, the high-P treatment with PSB inoculation under normal irrigation produced significantly higher DM (*p* < 0.05), with growth increments ranging from 16.10% to 39.67%.

### 3.6. Correlation Analysis of Alfalfa Parameters Under Different Treatments

**Figure 6:**
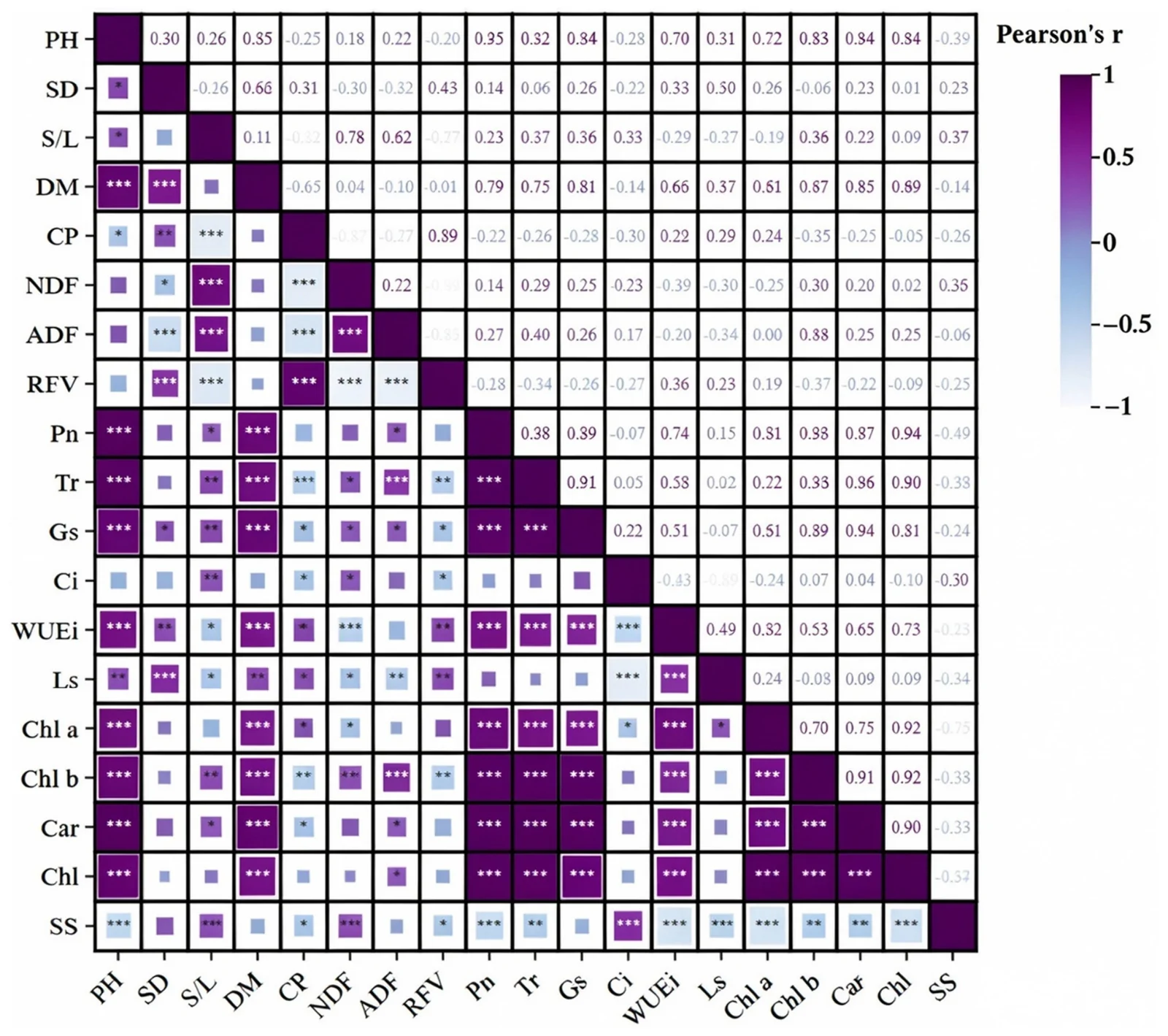
Correlation analysis of photosynthesis, nutritional quality, and growth performance of alfalfa under different treatments. Square size indicates the significance level, and color denotes positive or negative correlations. *p*-values less than 0.05, 0.01, and 0.001 are marked with asterisks “*”, “**”, and “***”, respectively. PH: plant height; SD: stem diameter; S/L: stem-to-leaf ratio; DM: dry matter yield; CP: crude protein content; NDF: neutral detergent fiber content; ADF: acid detergent fiber content; RFV: relative feed value; Pn: net photosynthetic rate; Tr: transpiration rate; Gs: stomatal conductance; Ci: intercellular CO_2_ concentration; WUEi: instantaneous water use efficiency; Ls: stomatal limitation value; Chl a: chlorophyll a content; Chl b: chlorophyll b content; Car: carotenoid content; Chl: total chlorophyll content; SS: soluble sugar content.

**Figure 7:**
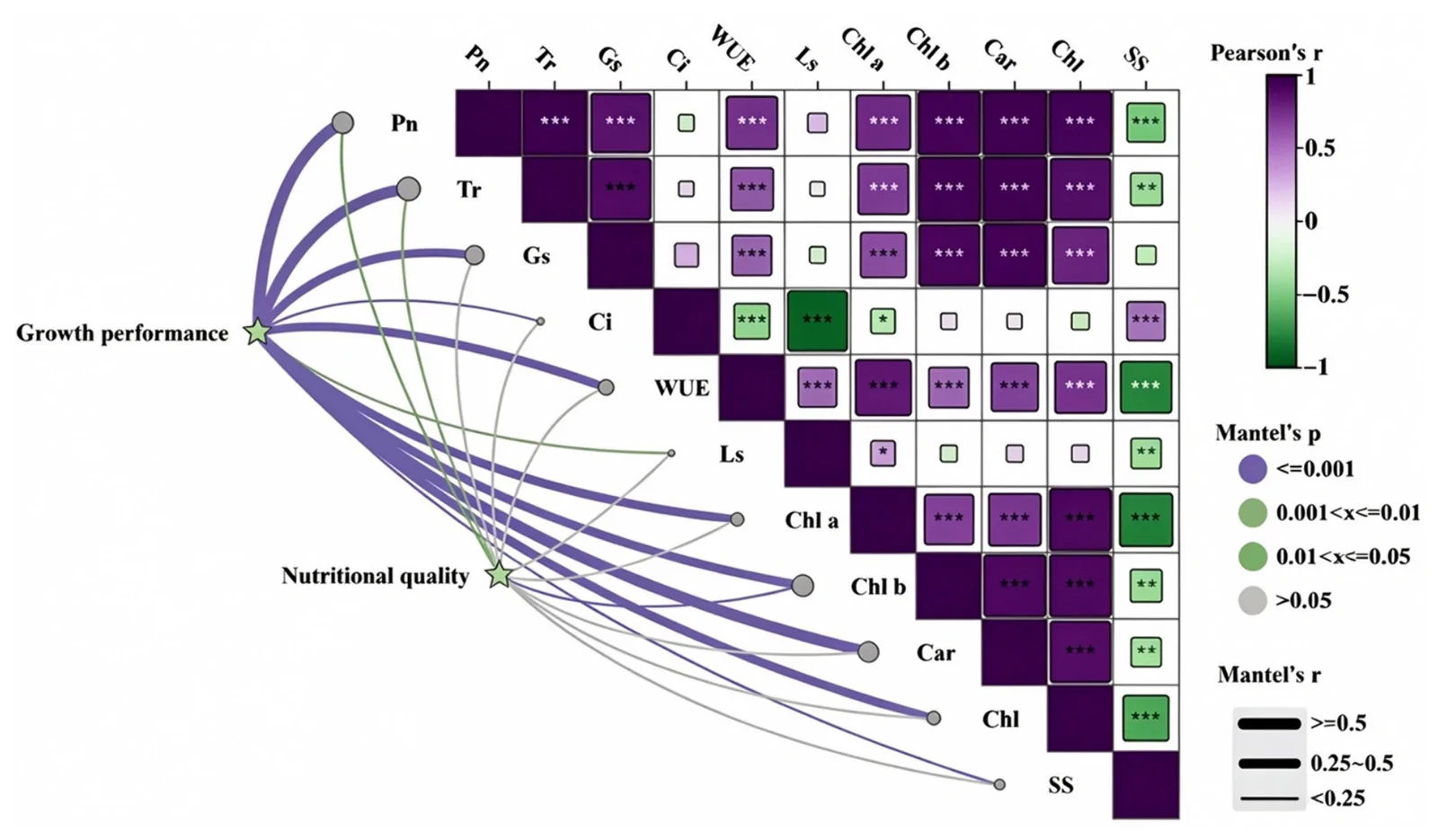
Mantel test correlation analysis illustrating the relationships among the growth performance, nutritional quality, and photosynthetic performance, Chl content, and SS content of alfalfa. Line thickness indicates the R^2^ level, square size represents the significance level, and square color denotes positive or negative correlations. *p*-values less than 0.05, 0.01, and 0.001 are marked with asterisks “*”, “**”, and “***”, respectively. Pn: net photosynthetic rate; Tr: transpiration rate; Gs: stomatal conductance; Ci: intercellular CO_2_ concentration; WUEi: instantaneous water use efficiency; Ls: stomatal limitation value; Chl a: chlorophyll a content; Chl b: chlorophyll b content; Car: carotenoid content; Chl: total chlorophyll content; SS: soluble sugar content.

To further investigate the relationships among photosynthesis, nutritional quality, and growth performance of alfalfa under water–phosphorus–microbial interactions, all biological replicate samples from 2024 and 2025 were pooled in this study for Pearson correlation analysis. In addition, Mantel tests were performed to evaluate the associations among different functional trait modules. The DM of alfalfa showed highly significant positive correlations with Pn, Tr, Gs, WUEi, Ls, Chl a content, Chl b content, Car content, and Chl (a + b) content (*p* < 0.01). The SS content of alfalfa was significantly negatively correlated with RFV, DM, CP content, ADF content, Pn, Tr, Gs, WUEi, and Chl (a + b) content (*p* < 0.05). RFV in alfalfa was significantly negatively correlated with Tr, Gs, Ci, and Chl b content, whereas it exhibited significant positive correlations with WUEi and Ls (*p* < 0.05) (Figure 6). The Mantel test further indicated that photosynthetic performance, chlorophyll content, and SS content of alfalfa were highly significantly correlated with growth performance and significantly positively correlated with nutritional quality (*p* < 0.05) (Figure 7).

### 3.7. Principal Component Analysis and Comprehensive Evaluation

**Figure 8:**
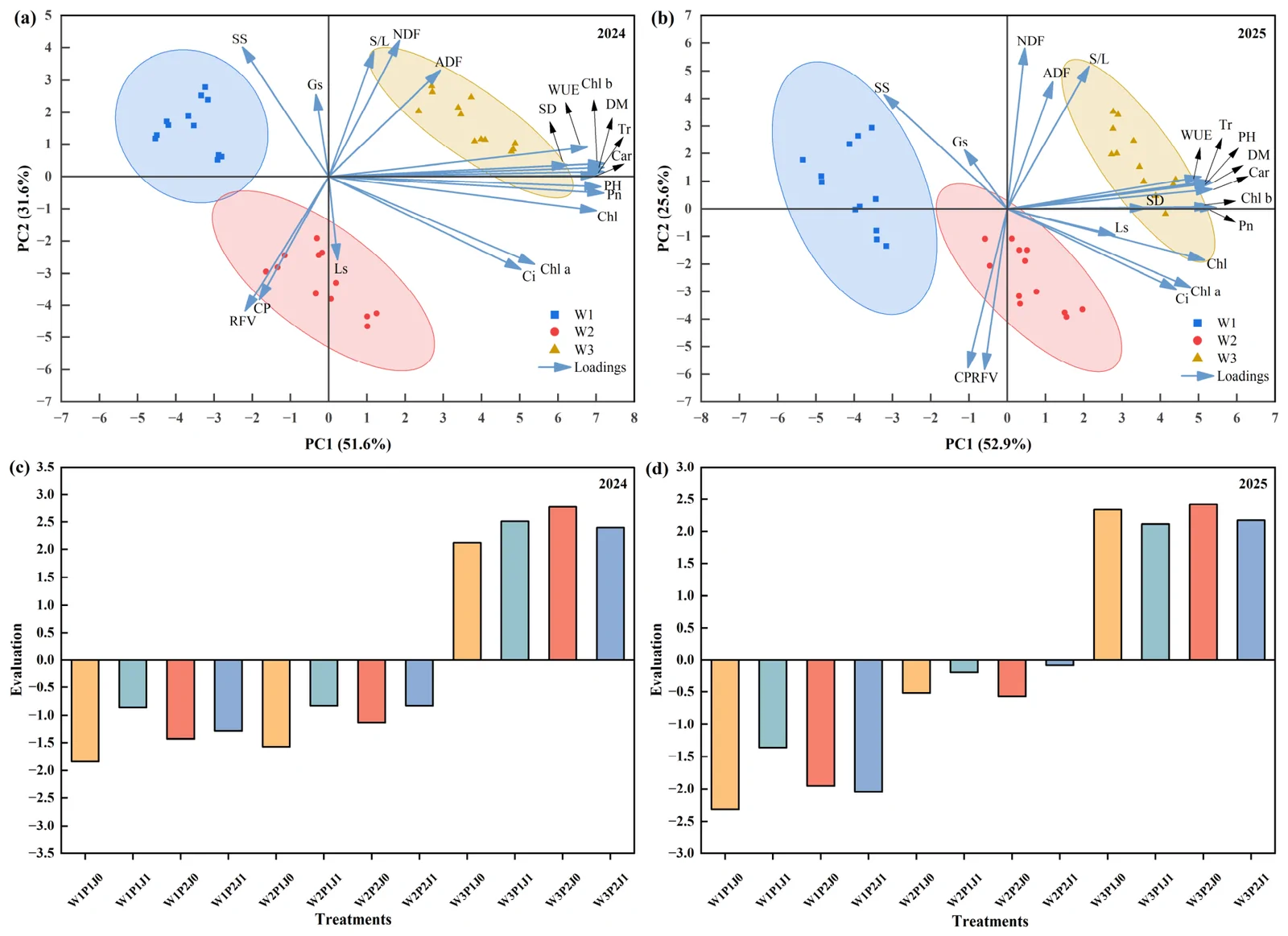
Principal component analysis (**a**,**b**) and comprehensive evaluation (**c**,**d**) of alfalfa photosynthesis, nutritional quality, and growth performance under water–phosphorus–bacteria interaction. (**a**,**b**) PCA ordination plots for 2024 and 2025, respectively, with the values in parentheses indicating the proportions of variance explained by PC1 and PC2. (**c**,**d**) Comprehensive evaluation results calculated based on principal component scores for 2024 and 2025, respectively. The four different colored bars within each water treatment represent P1J0, P1J1, P2J0 and P2J1, respectively. PH: plant height; SD: stem diameter; S/L: stem-to-leaf ratio; DM: dry matter yield; CP: crude protein content; NDF: neutral detergent fiber content; ADF: acid detergent fiber content; RFV: relative feed value; Pn: net photosynthetic rate; Tr: transpiration rate; Gs: stomatal conductance; Ci: intercellular CO_2_ concentration; WUEi: instantaneous water use efficiency; Ls: stomatal limitation value; Chl a: chlorophyll a content; Chl b: chlorophyll b content; Car: carotenoid content; Chl: total chlorophyll content; SS: soluble sugar content. W1, W2, and W3 represent moderate drought (40–45% field capacity), mild drought (55–60% field capacity), and normal irrigation (70–75% field capacity), respectively. P1 and P2 represent low P (50 mg kg^−1^ P_2_O_5_) and high P (100 mg kg^−1^ P_2_O_5_), respectively, while J0 and J1 represent non-inoculation (0 mL pot^−1^) and inoculation with PSB (10 mL pot^−1^), respectively.

Principal component analysis and comprehensive evaluation of photosynthesis, nutritional quality, and growth performance of alfalfa under the water–phosphorus–bacteria interaction are presented in Figure 8. Principal component analysis extracts principal components with eigenvalues greater than 1 and cumulative contribution rates exceeding 75%, which can reflect most of the information in the original data. The analysis showed that the contribution rates of PC1 and PC2 were 51.6% and 31.6%, respectively, in 2024, with a cumulative contribution rate of 83.2%; in 2025, the contribution rates of PC1 and PC2 were 52.9% and 25.6%, respectively, with a cumulative contribution rate of 78.5%. In terms of variable loadings, PC1 in both 2024 and 2025 showed high positive loadings for NDF, ADF, S/L, WUEi, Tr, DM, PH, Chl, Chl a, Chl b, Car, and Ci, but negative loadings for SS, CP, and RFV, indicating that PC1 is mainly composed of multiple variables related to alfalfa growth, photosynthesis, and forage quality, reflecting the synergistic variation in the above-mentioned indicators. PC2 mainly reflected the separation trend between indicators such as SS, Gs, NDF, ADF, and S/L and indicators such as CP, RFV, Ls, Chl, Chl a, and Ci. To a certain extent, it can characterize osmotic-adjustment and stomatal-response characteristics under drought stress. The loading structure in 2025 was generally consistent with that in 2024, indicating a certain degree of stability in the comprehensive evaluation results. The comprehensive PCA scores differed significantly among treatments. The overall trend in comprehensive scores was generally consistent between the two years, with the normally irrigated treatments showing significantly higher comprehensive scores than the drought-stress treatments. Under normal irrigation conditions, the influence of P fertilizer level and PSB inoculation on comprehensive performance was relatively minor. Under normal irrigation, the W3P2J0 and W3P1J1 treatments had the highest comprehensive scores. Under mild drought stress, treatments inoculated with PSB, including W2P1J1 and W2P2J1, generally performed better than the non-inoculated treatments, including W2P1J0 and W2P2J0. Under moderate drought stress, all treatments had relatively low comprehensive scores, regardless of P fertilizer level or PSB inoculation (Figure 8).

## 4. Discussion

### 4.1. Effects of Water–Phosphorus–Bacteria Interaction on the Growth Performance of Alfalfa

Drought stress is a major abiotic stress that constrains the growth and development of alfalfa, severely limiting plant production and causing substantial reductions in crop yield [23]. P and PSB act as key factors regulating plant growth, and their synergistic regulatory mechanism is critical to the growth and development of alfalfa in arid and semi-arid regions [19]. Research has shown that drought stress reduces soil moisture and limits the water available for plant growth, thereby impeding growth and reducing productivity [24]. This study demonstrates that under identical conditions of P fertilizer and PSB interaction, as the level of drought stress increased, the PH, SD, and DM of alfalfa gradually decreased, while the S/L showed an initial decline followed by an increase. These results indicate that drought stress reduces the production performance of alfalfa, reflecting the direct inhibitory effect of water deficit on cell expansion and photosynthetic carbon assimilation in plants [25]. However, under mild to moderate drought stress, the treatment combining high P levels with PSB significantly increased alfalfa PH and SD. This indicates that the combination of PSB and sufficient P sources can effectively alleviate the growth inhibition caused by drought stress. Previous studies have shown that PSB have P-solubilizing and growth-promoting potential and can promote the normal growth and development of alfalfa by mobilizing insoluble P in soil [19]. These two pathways synergistically achieve the dual effects of “drought resistance and growth promotion”. Under drought stress, the S/L of all treatments was lower than that under normal irrigation, mainly due to delayed maturity and the fact that water stress inhibits stem growth more significantly than leaf growth [26]. The minimum S/L over both years was observed in the W2P2J0 and W2P2J1 treatment groups. A lower S/L indicates a higher leaf proportion and potential forage value, suggesting that under mild drought stress, the application of high P combined with PSB not only alleviates the impact of drought stress on alfalfa growth but also optimizes biomass allocation by promoting the distribution of assimilates to the leaves, thereby improving forage quality [3]. This study showed that, under mild drought stress, SD and DM in the high-P plus PSB inoculation treatment were not significantly different from those in the low-P treatment under normal irrigation. This suggests that high P application combined with PSB inoculation can reduce the yield gap between mild drought stress and normal irrigation conditions and alleviate drought-induced growth inhibition to improve plant performance under drought environments**.** Nevertheless, this treatment only represents a stress-adaptive advantage under water-deficit conditions and does not achieve maximization of overall productivity.

### 4.2. Effects of Water–Phosphorus–Bacteria Interaction on Photosynthetic Pigment and Soluble Sugar Contents of Alfalfa

The content of photosynthetic pigments and the accumulation of SS are key physiological indicators reflecting the photosynthetic capacity and stress response of alfalfa. Studies have shown that photosynthetic pigments, as essential components of plant photosynthesis, play a critical role in light capture and energy conversion [27]. SS are important compounds involved in plant osmotic regulation and carbon storage, and their accumulation levels can reflect physiological responses of plants to drought stress. Meanwhile, SS content is also jointly regulated by multiple processes, including respiratory metabolic consumption and utilization for plant growth [28]. Under drought stress, inhibited plant growth and restricted carbon transport and utilization may suppress sugar metabolism, leading to an increase in SS content and thereby enhancing plant drought tolerance [29]. This study showed that SS content was highest under the moderate drought, low-P, non-inoculated treatment, whereas the moderate drought, high-P, PSB-inoculated treatment exhibited a moderate SS level and a relatively high RFV. These results suggest that severe drought stress can trigger SS accumulation in plants. Studies have shown that P is involved in regulating the synthesis of chlorophyll precursors, such as δ-aminolevulinic acid, and that PSB may affect leaf photosynthetic function by increasing P availability [30]. This study demonstrated that irrigation, P fertilizer, PSB, and all their interactions exerted extremely significant effects on the photosynthetic pigment contents and SS content of alfalfa. This indicates that water–phosphorus–bacteria interaction enhances the adaptability of alfalfa to drought environments by optimizing its physiological metabolism. Studies have shown that the maintenance of relatively high chlorophyll levels under mild drought stress facilitates photosynthetic carbon assimilation to meet growth requirements. With the intensification of drought severity, plant osmolytes such as proline, SS, and proteins contribute to osmotic adjustment, enabling plants to retain water and tolerate drought stress [31]. Previous studies have shown that, under mild drought stress, high P application combined with PSB inoculation may help maintain greater light-harvesting capacity and higher leaf photosynthetic pigment levels, thereby alleviating drought-induced inhibition of photosynthesis and promoting photosynthetic carbon fixation and plant growth [19]. A phosphorus application rate of 100 mg kg^−1^ P_2_O_5_ combined with mixed bacterial inoculation can improve photosynthetic efficiency and chlorophyll content in alfalfa leaves and promote plant growth [32]. This study demonstrated that under the synergistic effect of mild drought, high P supply, and PSB inoculation, alfalfa maintained relatively high photosynthetic pigment levels and a moderate SS content. Together with the enhanced photosynthetic performance, DM, and nutritional quality observed under this treatment, these findings indicate that photosynthetic products were maintained in a relatively coordinated state among osmotic regulation, growth maintenance, and quality formation [33]. Thus, under mild drought, the combined application of high P and PSB not only maintains photosynthetic efficiency by increasing photosynthetic pigment contents, but also helps coordinate the relationships among photosynthetic carbon assimilation, SS accumulation, and growth utilization, thereby inducing favorable photosynthetic physiological and quality-related responses.

### 4.3. Effects of Water–Phosphorus–Bacteria Interaction on Photosynthetic Characteristics of Alfalfa

Photosynthesis is a unique physiological function of green plants and serves as the material and energy foundation for crop yield formation and quality improvement. Enhancing photosynthesis plays a vital role in promoting crop dry matter accumulation [11]. This study demonstrated that under the same conditions of P fertilizer combined with PSB inoculation, with the intensification of drought stress severity, the Pn, Tr, and Gs of alfalfa all exhibited a continuous downward trend, while the Ls first increased and then decreased. This is consistent with the adaptive mechanism of plants to limit water loss through stomatal closure under water deficit conditions [34]. Under mild drought stress, the increase in WUEi was primarily attributable to a greater decrease in Tr than in Pn, indicating that plants reduced water loss through stomatal regulation, thereby enhancing carbon assimilation per unit of water consumed [35]. Under mild drought stress, Pn, Gs, and Ci decreased simultaneously, which may be attributed to insufficient CO_2_ supply caused by stomatal closure under mild drought conditions [34]. In contrast, under moderate drought stress, Ci was significantly higher than under normal irrigation, while Pn was lower, which may be associated with non-stomatal limitation processes at the mesophyll level under moderate drought stress [36]. This study showed that high P levels and PSB inoculation improved photosynthetic parameters, such as Pn and WUEi, under mild drought stress. Previous studies have demonstrated that P is involved in photosynthetic metabolic processes, including ATP and NADPH formation and the Calvin cycle; thus, improved P supply may help maintain photosynthetic carbon assimilation capacity [37]. Additionally, substances such as plant hormones and ACC deaminase produced by PSB can regulate the balance of plant endogenous hormones, optimize the coordination between stomatal behavior and carbon assimilation, thereby maintaining a relatively high photosynthetic rate while reducing water loss [33]. This study showed that under mild drought stress, the treatment with high P application combined with PSB inoculation achieved the highest WUEi. This may be because mild drought stress reduced water consumption by inducing stomatal closure and thereby decreasing the transpiration rate, thereby improving plant photosynthesis [38]. This indicates that mild drought stress reduces water consumption by inducing stomatal closure to decrease Tr, while high P supply and the phosphate-solubilizing ability of PSB synergistically ensure rhizosphere P availability, maintaining sufficient P to sustain a relatively high Pn. These two factors collectively enhance water use efficiency [38]. Thus, the water–phosphorus–bacteria interaction significantly affects the photosynthetic characteristics of alfalfa by regulating stomatal behavior, mesophyll photosynthetic capacity, and water use efficiency.

### 4.4. Effects of Water–Phosphorus–Bacteria Interaction on Nutritional Quality of Alfalfa

Plants regulate organ traits through phenotypic plasticity to achieve optimal resource allocation, among which preferential allocation to leaves is a key adaptive strategy for improving forage nutritional quality [39]. CP, NDF, ADF, and RFV are important indices for evaluating the nutritional quality of alfalfa [40]. Studies have shown that mild water deficit induces plants to initiate stress adaptation responses, whereby plants redistribute photosynthetic carbon and metabolic energy to direct more resources toward the biosynthesis of nitrogen-containing compounds for osmotic adjustment; accordingly, the synthesis of cell wall structural carbohydrates is inhibited to a certain extent [25]. This study demonstrated that irrigation, P application, and PSB inoculation exerted extremely significant effects on the nutritional quality of alfalfa. This indicates that phosphorus nutrition plays a crucial role in alfalfa production; previous studies have shown that inoculation with Arbuscular Mycorrhizal Fungi (AMF) and PSB can improve the nutritional quality of alfalfa [19]. Additionally, an appropriate P fertilizer level promotes protein synthesis in alfalfa, inhibits the excessive accumulation of structural carbohydrates, and thereby synergistically enhances both DM and nutritional quality of alfalfa [41]. Simultaneously, most phosphate-solubilizing strains can alleviate the negative impacts of drought stress on plant metabolism through mechanisms such as producing osmotic adjustment substances [42]. This study demonstrated that under the same conditions of P fertilizer combined with PSB inoculation, compared with the normal irrigation treatment, drought stress resulted in increased CP content and decreased NDF and ADF levels. The primary reason is that the S/L of alfalfa was lower under drought stress than under normal irrigation, leading to an increased proportion of leaves in the plant and thereby enhancing the CP content under drought stress [43]. Studies have shown that drought stress inhibits the growth rate of maize, prolongs the vegetative growth stage, alters root direction, and redistributes the translocation and distribution of photosynthates [44]. Studies have shown that PSB can secrete organic acids to mobilize sparingly soluble P in soil and optimize root uptake capacity, thereby simultaneously enhancing plant nitrogen and P absorption [45]. This study shows that under mild drought stress, the combined application of high P fertilizer and PSB can simultaneously maximize CP content while minimizing the levels of NDF and ADF in alfalfa. RFV is a key indicator for comprehensive evaluation of forage nutritional quality. An increase in RFV signifies a notable improvement in the potential intake and energy value of alfalfa as a feed [46].

## 5. Conclusions

The integrated water–phosphorus–bacteria management strategy effectively mitigates the inhibitory effect of drought stress on alfalfa, while synergistically improving its productivity and nutritional quality by optimizing photosynthetic characteristics and the allocation of photoassimilates. Under the present pot experimental conditions, combined high-P application and PSB inoculation under mild drought stress could alleviate the adverse effects of drought stress on plant growth and development. This treatment maintained alfalfa DM at a level comparable to that of the low-P treatment under normal irrigation, and reduced the S/L, thus optimizing plant biomass allocation characteristics. It sustained relatively high chlorophyll content and Pn, significantly improved WUEi, and enhanced the photosynthetic performance of alfalfa under drought stress. In terms of forage quality, this treatment markedly increased CP content and decreased NDF and ADF. Compared with normal-irrigation treatments, the RFV of the W2P2J1 treatment increased by 15.75–15.81%, showing obvious improvement in forage quality. In summary, the combination of mild drought stress (55–60% of field capacity), a high-P level (P_2_O_5_ 100 mg kg^−1^), and inoculation with the PSB strain used in this study enabled alfalfa to achieve superior photosynthetic traits, water-use efficiency, and forage quality, showing application potential for water-saving and quality-improving alfalfa cultivation in arid and semi-arid regions. Given that this was a controlled pot experiment, this management mode still requires further validation under non-sterilized soil and field conditions. This study provides a theoretical reference for water-saving, quality-improving, and efficiency-enhancing production of alfalfa in arid and semi-arid regions.

## Figures and Tables

**Table 1:** Physical and chemical properties of the tested soil.

Organic Matter	Bulk Density	Total Nitrogen	Total Phosphorus	Alkaline Nitrogen	Available Phosphorus	Available Potassium
(g·kg^−1^)	(g·cm^−3^)	(g·kg^−1^)	(g·kg^−1^)	(mg·kg^−1^)	(mg·kg^−1^)	(mg·kg^−1^)
23.60	1.47	1.55	0.22	70.50	18.10	135.20

**Table 2:** Different irrigation, phosphorus application levels, and strain inoculation treatments.

Treatment	Irrigation (Field Capacity %)	Phosphorus Fertilizer (mg kg^−1^ P_2_O_5_)	*Bacillus mucilaginosus* (mL pot^−1^)
W1P1J0	40–45	50	0
W1P1J1	40–45	50	10
W1P2J0	40–45	100	0
W1P2J1	40–45	100	10
W2P1J0	55–60	50	0
W2P1J1	55–60	50	10
W2P2J0	55–60	100	0
W2P2J1	55–60	100	10
W3P1J0	70–75	50	0
W3P1J1	70–75	50	10
W3P2J0	70–75	100	0
W3P2J1	70–75	100	10

Note: W1, W2, and W3 represent moderate drought (40–45% of field capacity), mild drought (55–60% of field capacity), and normal irrigation (70–75% of field capacity), respectively; P1 and P2 represent low phosphorus (50 mg kg^−1^ P_2_O_5_) and high phosphorus (100 mg kg^−1^ P_2_O_5_), respectively; and J0 and J1 represent non-inoculation (0 mL pot^−1^) and PSB inoculation (10 mL pot^−1^), respectively.

## Data Availability

The raw data supporting the conclusions of this article will be made available by the authors on request.

## Supplementary Materials

The following supporting information can be downloaded at https://www.mdpi.com/article/10.3390/biology15201903/s1, Table S1: Variance analysis of irrigation, phosphate fertilizer, phosphate-solubilizing bacteria and their interaction on alfalfa growth traits; Table S2: Variance analysis of irrigation, phosphate fertilizer and phosphate-solubilizing bacteria and their interaction on chlorophyll and soluble sugar content of alfalfa; Table S3: Variance analysis of irrigation, phosphate fertilizer, phosphate-solubilizing bacteria and their interaction on photosynthetic characteristics of alfalfa; Table S4: Analysis of variance of irrigation, phosphate fertilizer, phosphate-solubilizing bacteria and their interaction on nutritional quality of alfalfa.
