# Supplementary material for: Irrigation, Phosphorus Application, and Inoculation Improve the Yield and Nutritional Quality of Alfalfa by Regulating Its Photosynthetic Performance
Source: Biology (Basel). 2026 Oct 21;15(20):1903. doi: 10.3390/biology15201903 (PMC13701999; doi:10.3390/biology15201903)
Supplement: Supplementary file 1 [file biology-15-01903-s001.zip › biology-4614393-supplementary.pdf]

Table S1 Variance analysis of irrigation, phosphate fertilizer, phosphate-solubilizing bacteria and their interaction on alfalfa growth traits

Source of variable	Year	Plant height		Stem diameter		Stem-to-leaf ratio		Dry matter yield	
		F	*P*	*F*	*P*	*F*	*P*	*F*	*P*
W	2024	893.46	0.00	94.10	0.00	253.68	0.00	806.83	0.00
P	2024	9.00	0.00	17.16	0.00	149.21	0.00	69.45	0.00
J	2024	16.48	0.00	10.25	0.00	4.61	0.04	104.03	0.00
W×P	2024	0.32	0.73	1.48	0.23	30.61	0.00	37.91	0.00
W×J	2024	4.93	0.01	3.09	0.05	4.67	0.02	1.11	0.35
P×J	2024	0.81	0.37	0.02	0.90	4.05	0.06	4.30	0.05
W×P×J	2024	3.09	0.05	0.48	0.62	21.78	0.00	16.33	0.00
W	2025	1040.90	0.00	26.77	0.00	179.54	0.00	1116.57	0.00
P	2025	13.26	0.00	14.71	0.00	11.39	0.00	5.98	0.02
J	2025	1.76	0.19	0.16	0.69	20.46	0.00	111.85	0.00
W×P	2025	2.03	0.14	0.05	0.96	4.24	0.03	12.41	0.00
W×J	2025	3.86	0.02	3.18	0.05	2.71	0.09	0.32	0.73
P×J	2025	3.29	0.07	3.74	0.06	20.46	0.00	2.38	0.14
W×P×J	2025	6.36	0.00	1.70	0.19	13.47	0.00	6.52	0.01

Note: W represents irrigation level; P represents phosphorus application level; J represents inoculation level.

Table S2 Variance analysis of irrigation, phosphate fertilizer and phosphate-solubilizing bacteria and their interaction on chlorophyll and soluble sugar content of alfalfa.

Source of variable	Year	Chlorophyll a		Chlorophyll b		Carotenoid		Chlorophyll a+b		Soluble sugar	
		F	*P*	*F*	*P*	*F*	*P*	*F*	*P*	*F*	*P*
W	2024	628.32	0.00	2860.75	0.00	445.54	0.00	1771.47	0.00	1794.93	0.00
P	2024	309.13	0.00	85.75	0.00	37.50	0.00	291.67	0.00	38.52	0.00
J	2024	212.00	0.00	150.89	0.00	32.67	0.00	272.01	0.00	13.41	0.00
W×P	2024	34.21	0.00	10.75	0.00	14.63	0.00	36.61	0.00	52.07	0.00
W×J	2024	8.85	0.00	3.25	0.06	1.54	0.23	9.75	0.00	1.04	0.37
P×J	2024	3.70	0.07	15.75	0.00	8.17	0.01	11.67	0.00	2.30	0.14
W×P×J	2024	4.55	0.02	2.68	0.09	2.79	0.08	4.84	0.02	6.57	0.01
W	2025	2978.73	0.00	1163.40	0.00	1175.27	0.00	3299.94	0.00	625.52	0.00
P	2025	622.23	0.00	140.45	0.00	129.07	0.00	620.89	0.00	52.71	0.00
J	2025	137.50	0.00	36.45	0.00	26.67	0.00	142.63	0.00	30.68	0.00
W×P	2025	39.82	0.00	38.60	0.00	8.87	0.00	65.37	0.00	54.35	0.00
W×J	2025	86.36	0.00	18.60	0.00	11.27	0.00	89.28	0.00	11.29	0.00
P×J	2025	33.14	0.00	22.05	0.00	6.67	0.02	61.07	0.00	0.19	0.67
W×P×J	2025	75.82	0.00	29.40	0.00	4.07	0.03	92.37	0.00	5.19	0.01

Note: W represents irrigation level; P represents phosphorus application level; J represents inoculation level.

Table S3 Variance analysis of irrigation, phosphate fertilizer, phosphate-solubilizing bacteria and their interaction on photosynthetic characteristics of alfalfa

Source of variable	Year	Net photosynthetic rate		Transpiration rate		Stomatal conductance		Intercellular CO_2 concentration		Instantaneous water use efficiency		Stomatal limitation value	
		F	*P*	*F*	*P*	*F*	*P*	*F*	*P*	*F*	*P*	*F*	*P*
W	2024	15336.29	0.00	1532.52	0.00	458.64	0.00	241.24	0.00	168.15	0.00	240.41	0.00
P	2024	1480.77	0.00	106.39	0.00	115.12	0.00	241.64	0.00	23.43	0.00	240.94	0.00
J	2024	251.96	0.00	19.67	0.00	0.75	0.40	14.07	0.00	3.53	0.07	15.06	0.00
W×P	2024	53.95	0.00	1.22	0.31	9.59	0.00	8.79	0.00	4.45	0.02	11.71	0.00
W×J	2024	97.69	0.00	6.94	0.00	9.22	0.00	4.59	0.02	1.75	0.20	6.41	0.01
P×J	2024	4.94	0.04	0.16	0.69	16.56	0.00	5.42	0.03	1.17	0.29	5.88	0.02
W×P×J	2024	114.58	0.00	12.60	0.00	6.58	0.01	5.19	0.01	0.12	0.89	4.29	0.03
W	2025	4367.98	0.00	1379.79	0.00	1794.81	0.00	487.68	0.00	122.7	0.0	256.72	0.00
P	2025	440.40	0.00	90.10	0.00	441.58	0.00	310.13	0.00	29.4	0.0	156.06	0.00
J	2025	51.34	0.00	17.15	0.00	3.90	0.06	56.15	0.00	1.1	0.3	20.06	0.00
W×P	2025	15.08	0.00	2.87	0.08	15.77	0.00	11.39	0.00	8.9	0.0	8.39	0.00
W×J	2025	29.28	0.00	7.54	0.00	25.39	0.00	3.51	0.05	1.6	0.2	11.06	0.00
P×J	2025	21.24	0.00	3.10	0.09	7.26	0.01	8.91	0.01	1.7	0.2	29.39	0.00
W×P×J	2025	12.57	0.00	3.76	0.04	10.74	0.00	29.73	0.00	0.8	0.5	6.72	0.01

Note: W represents irrigation level; P represents phosphorus application level; J represents inoculation level.

Table S4 Analysis of variance of irrigation, phosphate fertilizer, phosphate-solubilizing bacteria and their interaction on nutritional quality of alfalfa

Source of variable	Year	Crude protein		Neutral detergent fiber		Acid detergent fiber		Relative feeding value	
		F	*P*	*F*	*P*	*F*	*P*	*F*	*P*
W	2024	150.44	0.00	63.12	0.00	78.13	0.00	80.63	0.00
P	2024	112.86	0.00	34.70	0.00	3.55	0.07	34.30	0.00
J	2024	5.35	0.03	15.97	0.00	40.55	0.00	33.81	0.00
W×P	2024	12.43	0.00	5.29	0.01	4.53	0.02	5.87	0.01
W×J	2024	2.36	0.12	20.41	0.00	11.04	0.00	17.30	0.00
P×J	2024	6.56	0.02	6.09	0.02	5.70	0.03	2.23	0.15
W×P×J	2024	16.16	0.00	11.45	0.00	5.45	0.01	6.34	0.01
W	2025	213.45	0.00	27.82	0.00	0.38	0.69	19.67	0.00
P	2025	124.51	0.00	132.21	0.00	95.31	0.00	159.13	0.00
J	2025	4.94	0.04	56.05	0.00	18.74	0.00	55.19	0.00
W×P	2025	14.96	0.00	29.06	0.00	1.59	0.23	25.42	0.00
W×J	2025	1.29	0.29	8.26	0.00	6.15	0.01	4.90	0.02
P×J	2025	4.51	0.04	0.73	0.40	23.09	0.00	2.75	0.11
W×P×J	2025	25.62	0.00	4.40	0.02	5.83	0.01	5.43	0.01

Note: W represents irrigation level; P represents phosphorus application level; J represents inoculation level.
